# Normative Values of Rhinology Questionnaires in Young Adults: A Tool to Identify Candidates for Rhinoplasty

**DOI:** 10.3390/life15020170

**Published:** 2025-01-24

**Authors:** Piotr Rot, Paweł Piotr Grab, Marta Kwiatkowska, Łukasz Skrzypiec, Sandra Krzywdzińska, Dariusz Jurkiewicz, Maria Sobol

**Affiliations:** 1 Department of Otolaryngology with Division of Cranio-Maxillo-Facial Surgery, Military Institute of Medicine—National Research Institute, Szaserów 128, 04-141 Warsaw, Poland; prot@wim.mil.pl (P.R.); mkwiatkowska1@wim.mil.pl (M.K.); lskrzypiec@wim.mil.pl (Ł.S.); djurkiewicz@wim.mil.pl (D.J.); 2 Clinical Department of Cranio-Maxillo-Facial Surgery, Military Institute of Medicine—National Research Institute, Szaserów 128, 04-141 Warsaw, Poland; pgrab@wim.mil.pl; 3 Department of Biophysics, Physiology and Pathophysiology, Medical University of Warsaw, 02-901 Warsaw, Poland

**Keywords:** FROI-17, young adults, nasal symptoms, normative values, rhinoplasty, ROE, questionnaire-based

## Abstract

Objective: Normative values of Rhinoplasty Outcome Evaluation (ROE) and Functional Rhinoplasty Outcome Inventory 17 (FROI-17) allow the monitoring of surgery outcomes. The objective of our study was to determine the reference norms of these disease-specific questionnaires in the age group that most often seeks rhinoplasty. Methods: The normative values of the ROE and FROI-17 questionnaires were calculated for 570 (459 women and 111 men) young adults at the mean age of 19.3 ± 1.3 years, range 18–25 years. Each participant underwent an ENT examination. All those who obtained a positive result were asked to complete two questionnaires: ROE and FROI-17. Results: The mean total ROE score was 13.4 ± 2.3, with a median of 13 and a range from 7 to 24. The mean overall FROI-17 score was 9.1 ± 13.3, with a median of 4 and a range from 4 to 72. For nasal symptoms, the mean was 4.0 ± 6.0, with a median of 1 and a range from 0 to 29. We observed a statistically significant difference between men and women only for the normative values of nasal symptoms (mean 4.0 ± 5.9 and median 2 (0–27) vs. mean 3.7 ± 6.5 and median 0 (0–29)). Additionally, there was a statistically significant correlation between the normative values of the ROE and FROI-17 scores (ρs = −0.413 for all participants, ρs = −0.314 for women, and ρs = −0.437 for men). Conclusions: The established normative values for the ROE and FROI-17 questionnaires among young, healthy individuals without nasal abnormalities can assist in the initial assessment of individuals seeking rhinoplasty. Deviations from these normative values in the ROE and FROI-17 questionnaires results may serve as indicators of potential concerns, such as body dysmorphic disorder (BDD).

## 1. Introduction

Rhinoplasty is one of the most common cosmetic surgical procedures. It is also one of the most challenging procedures due to numerous variables that impact the functional and aesthetic outcomes [1,2,3]. As such, rhinoplasty patients are more likely to be less satisfied with the end result of surgery compared to other facial cosmetic procedures [4,5].

Patient satisfaction and improved quality of life (QoL) should be an important element of determining the outcomes of rhinoplasty and septorhinoplasty. Therefore, Alsarraf introduced outcomes research to facial plastic surgery in an effort to quantify patients’ subjective opinions [6]. Since that time, several patient-reported outcome measures have been developed. However, the Rhinoplasty Outcome Assessment (ROE) is the QoL questionnaire of choice for the majority of clinicians [7]. The ROE contains six questions, with two questions assessing each category: physical, emotional, and social factors of patient satisfaction with the procedure. The answers to each question are provided on a 5-point Likert scale [7,8,9].

The ROE is perceived as easy to use and was translated and validated into languages other than English: Arabic [10], Brazilian-Portuguese [11], German [12], Persian [4], Spanish [13], and Turkish [14]. Meta-analyses of the ROE demonstrated that it was a beneficial PROM in assessing the QoL after rhinoplasty. A statistically significant difference was found between ROE scores before and after surgical procedures [5]. Another meta-analysis highlighted the importance of improving ROE according to specific regions and cultures [15]. The authors of a meta-analysis of 16 studies of the QoL after functional rhinoplasty concluded that there was a statistically significant increase in the QoL based on the ROE score [16].

The limitation of the ROE is that it focuses on the aesthetic factors of the QoL and does not include the QoL-related functional aspects after septorhinoplasty. In 2014, Bulut et al. developed the FROI-17 with the aim of evaluating both the functional and aesthetic outcomes [17]. The FROI-17 has 17 items, ranging from physical symptoms, such as “constantly running nose” and “dry throat”, to emotional issues, such as “irritability” and “low self-esteem”. Each of the symptoms is assessed using a 6-point Likert scale, where 0 indicates no problem and 5 means the worst problem possible. The questionnaire takes approximately 5–10 min to complete [17,18].

The aim of this study was to provide the first normative values for the ROE and FROI-17 questionnaires for young adults whose health status was assessed by an ENT specialist. Normative values of the ROE and FROI-17 allow the monitoring of surgery outcomes. The determination of norms of these disease-specific questionnaires in the age group that most often seeks rhinoplasty is essential to determine good candidates for the QoL improvement after surgery.

## 2. Materials and Methods

The study was conducted in 2024 on 570 (459 women and 111 men) young adults at the mean age of 19.6 ± 3.0 years, ranging from 18 to 25 years. Patients were referred to an outpatient clinic where they underwent evaluations confirming their rhinological health. During the examination, the respondents received surveys containing the ROE questionnaire and the FROI-17 questionnaire, which they completed independently. All of them found themselves healthy, and during the rhinological examination, there were no pathological findings observed. Conscious agreement was obtained from all individual study participants. The research was approved by The Ethical Committee of the Military Medical Chamber, no. KB/41/24.

The inclusion criteria were age between 18 and 25 years, a negative history of nasal and paranasal sinus disorders, and no abnormalities in the rhinological examination.

The exclusion criteria were diseases of the nose and/or paranasal sinuses, a history of depression (from patient history), and a lack of consent to participate in the study.

During the study, 8 patients (6 men and 2 women) were excluded due to the inaccurate completion of the ROE and/or FROI-17 questionnaires.

The data were statistically analyzed.

## 3. Statistical Analysis

Statistical analysis was performed using the Statistica 13.1 package. In the computation of the sample size, we assumed a dependent sample *t*-test. The calculations were based on the results of a previous study by Plath et al. [18], with the assumption that all the responses within a subject group are normally distributed. Moreover, the calculations were made with a minimum of 80% power and a significance level of 5%. With these assumptions, a sample size of 418 patients would yield a power >80% and statistically significant pair differences of 95% confidence. The quantitative variables were summarized using descriptive statistics: mean, standard deviation, median, and range (Table 1 and Table 2). The distribution of each quantitative variable was tested for consistency with a normal distribution using the Shapiro–Wilk test. The categorical data were presented as frequencies and percentages. To assess differences between groups according to the sex for the continuous variables, the nonparametric Mann–Whitney *U* test was performed, as the variables were not normally distributed (Table 3). Correlation analysis was conducted using the Spearman’s correlation coefficient (Table 4). Statistical significance was set at the *p*-value < 0.05.

## 4. Results

The normative values of the ROE and FROI-17 questionnaires were calculated for all participants and for deviation according to the sex. Moreover, in the case of the FROI-17 questionnaires, the scores for each subscale, i.e., nasal symptoms, general symptoms, and self-confidence, were calculated apart from the overall score (Table 1).

**Figure 1:**
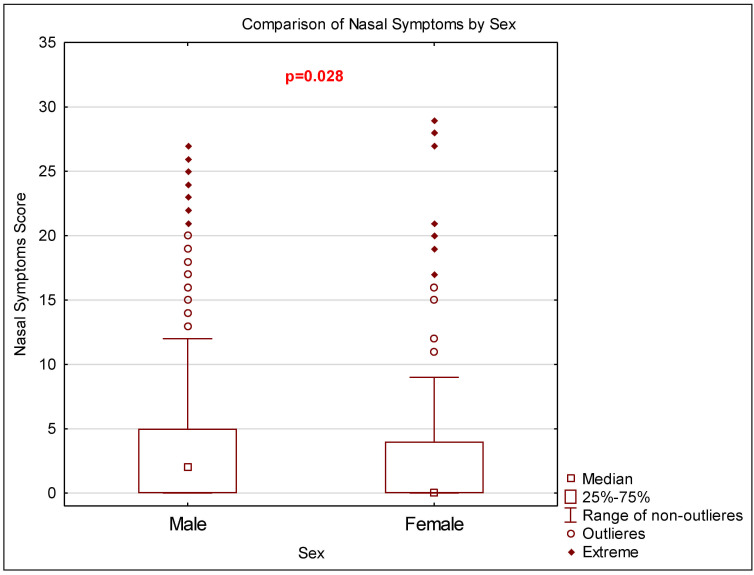
Sex-based differences in nasal symptoms.

For nasal symptoms, we noted statistically significant differences regarding sex (Figure 1). The median of the nasal symptoms was significantly lower in women than in men. The observations were similar for general symptoms, but the result was not statistically significant. Moreover, for the overall scores of ROE and FROI-17, as well as self-confidence, we did not notice statistically significant differences.

We noted a significant correlation between the total ROE and FROI-17 scores in all participants, as well as separately for both men and women (Table 3). For all participants, the Spearman’s rank correlation coefficient indicated a moderate negative correlation (ρ = −0.413). Similarly, a moderate negative correlation was observed in men (ρ = −0.437). In contrast, we observed a weak negative correlation in women, with a Spearman’s coefficient of ρ = −0.314 (Table 4).

## 5. Discussion

Rhinoplasty is one of the most popular cosmetic surgical procedures, especially among younger individuals [19,20]. Given this high demand, it becomes crucial to understand the normative values of the ROE and FROI-17 scores for this demographic. These standardized questionnaires play a key role in clinical practice, as highlighted by Wahmann et al. in 2018 [7], and are widely recognized as the most frequently used tools for assessing both the patient’s satisfaction with the appearance of their nose and the physical functionality of their nasal structures.

Moreover, since age is a significant factor influencing both the aesthetic and functional outcomes of rhinoplasty, it is essential to establish normative values specific to the age group that most commonly undergoes the procedure. Younger patients, for instance, may have different expectations and anatomical considerations compared to older individuals. Therefore, our aim was to provide these normative values for ROE and FROI-17 scores, encompassing not just the overall outcome but also the specific components, such as nasal symptoms, general health symptoms, and self-confidence levels.

Nasal diseases could affect both the structural and functional aspects of the nose, possibly influencing the effectiveness of aesthetic assessments or surgical outcomes. Similarly, individuals with depression may have different expectations or psychological responses to surgical results, which could affect both subjective satisfaction and the reliability of postoperative evaluations. Previous studies by Skwirczyńska et al. and Bulut et al. showed that mental health impairments might significantly impact physical health, particularly in young and healthy cohorts [21,22]. By clarifying these exclusion criteria, authors can provide a clearer rationale for their sample selection, helping to ensure that study findings are relevant and applicable to a healthy population without confounding factors.

The results of numerous meta-analyses have consistently shown significant improvements in both ROE and FROI-17 scores after rhinoplasty, with a notable increase in ROE scores post-treatment, indicating higher levels of patient satisfaction. Such an improvement reinforces the value of rhinoplasty in enhancing both the appearance and function. Additionally, patients who score below the normative threshold preoperatively are typically considered good candidates for surgery, as they are likely to experience the most substantial benefits from the procedure. These data also provide an important consideration for insurance companies as a compelling argument for the reimbursement of rhinoplasty procedures when they are shown to significantly improve the patient’s quality of life and nasal function [5]. Understanding the normative values is important, and broader implications for patient care and insurance consideration are highlighted.

In 2021, the normative values of the ROE and FROI-17 questionnaires were provided for the first time. Plath et al. analyzed a sample of 1000 German individuals recruited via a non-probability online panel [18]. However, the participants of this study did not undergo an ENT examination; they were recruited online, so they were reported healthy by themselves. Then, the mean age of the group was 44.3 ± 14.2 years, with the ages ranging from 18 to 69 years, which is quite an old population in context of rhinoplasty. Plath et al. found that age and sex significantly affected the overall FROI score and self-confidence scores (*p* < 0.05). They also noted that only age significantly influenced the ROE score. Additionally, Arima et al. reported that patients younger than 30 years had lower satisfaction improvements compared to those over 30 following crooked nose surgery [23]. Additionally, Schwitzer et al. found that rhinoplasty patients younger than 35 were more likely to experience enhanced satisfaction with the QoL compared to those older than 35 years [24]. Therefore, it is crucial to know the normative values of these questionnaires across different age groups and to consider sex differences.

In contrast to Plath et al. [18], we found no statistically significant differences regarding the overall ROE and FROI-17 scores according to sex (*p* = 0.908 and *p* = 0.220, respectively). In our study, the overall FROI-17 scores were comparable in men and women and notably lower than those reported by Plath et al. (22.4 ± 17.1 vs. 9.4 ± 12.9 for women and 19.1 ± 17.0 vs. 9.2 ± 12.9 for men). Moreover, we observed statistically significant differences in nasal symptoms, i.e., women had a lower median score than men but showed a wider range of variability (0–29 for women and 0–27 for men). A better perception of nasal symptoms in women may be associated with the fact that male gender is a negative predictor for satisfaction in patients seeking facial cosmetic surgery [25]. Similar to the overall scores in each subscale, we received much lower results compared to Plath et al. The differences between our results and those of Plath et al. may be influenced by the fact that we considered a younger age group.

Our study revealed a statistically significant correlation between the ROE and FROI-17 scores. The Spearman’s correlation coefficient was lower for men than women (−0.437 vs. −0.314). Bulut et al. examined patients who underwent septorhinoplasty [17]. To assess the QoL after surgery, patients were asked to complete the FROI-17, ROE, and SF-36 (Short Form 36 Health Survey) questionnaires. In those studies, the authors calculated correlations between the FROI-17, ROE, and SF-36 scores. The reported correlation analysis between SF-36 and both FROI-17 and ROE showed a statistically significant association only between FROI-17 and SF-36. The observed preoperative correlation was stronger than the postoperative one. However, the correlation between FROI-17 and ROE was not investigated in the studies.

As highlighted in the literature, conditions such as body dysmorphic disorder (BDD) and borderline personality disorder (BPD) are prevalent in up to 15% of patients seeking aesthetic procedures, including rhinoplasty and other cosmetic surgeries. These psychological conditions may significantly influence a patient’s motivation for seeking surgery and their overall satisfaction with the results. In such cases, performing surgery alone is unlikely to yield successful outcomes, as the underlying psychological issues may remain unaddressed. Patients with BDD, for instance, tend to have a distorted perception of their appearance, focusing excessively on minor or imagined flaws. Meanwhile, those with BPD often experience emotional instability and may have unrealistic expectations, leading to dissatisfaction with surgical results, even if the procedure is technically flawless [26].

In this context, we believe that incorporating normative data into the preoperative evaluation process may serve as a valuable tool for identifying potential psychological concerns early on. By comparing patients’ self-reported satisfaction and expectations (as measured by tools such as the ROE and FROI-17) with established normative values, clinicians may be able to detect discrepancies between objective assessments and subjective perceptions. For instance, if a patient’s self-evaluation reflects a significantly lower score compared to the normative data, despite there being no evident anatomical or functional issues, it may signal the presence of psychological conditions such as BDD or BPD. Such a discrepancy could indicate that the patient’s dissatisfaction is rooted in a psychological disorder rather than an actual physical defect. Validated tools, such as FROI-17 and ROE, should become standard components of clinical workflows to help with identify patients with unrealistic expectations or underlying psychological conditions. The questionaries are generally derived from broad populations that may not fully account for the influence of psychological conditions. These scores, however, might exhibit distinct patterns in patients with such conditions, influencing both their perception of outcomes and their satisfaction with surgery. Identifying such patterns during preoperative assessments could enable clinicians to refer these patients for further psychological evaluation before surgery. Postoperative outcomes in patients with psychological conditions might also differ from those in the general population, often showing a less significant improvement in satisfaction or quality of life [27,28]. Recognizing these trends can help refine metrics for surgical success and manage patient expectations more effectively. Research exploring targeted interventions, such as preoperative psychological counseling or psychological therapy, could play a crucial role in aligning postoperative outcomes with patient satisfaction. Furthermore, these findings could have implications for insurance reimbursement policies. Patients could have mandated psychological screenings for elective rhinoplasty, particularly in cases where psychological concerns are identified during initial consultations. This requirement would ensure that surgeries are pursued for the right reasons, increasing the likelihood of positive postoperative outcomes and significant improvements in patients’ quality of life [29,30].

By integrating objective measurements and psychological evaluations into clinical practice, a more comprehensive approach to rhinoplasty can be developed. Understanding how normative scores vary in populations with psychological conditions is crucial for advancing patient-centered care. Incorporating these insights into future research and clinical workflows will help clinicians better identify, support, and treat patients whose aesthetic concerns are closely tied to their psychological well-being. Ultimately, in our opinion, these advancements would refine clinical decision-making criteria and contribute to more equitable and effective patient care. In line with Iulian-Alexandru Taciuc et al. we are still far from using neural networks in otolaryngology, mainly because of a lack of a baseline for training [31].

Determining how normative values change over time following rhinoplasty seems essential for assessing long-term effects. Plat et al. did not notice significant differences between the normative ROE and FROI-17 scores and those of septorhinoplasty patients at 6 and 12 months after surgery to the reference cohort, except for the FROI-17 general score at 12 months after surgery (72.4 vs. 68.5) [18]. We do not have a lot of data on that topic in the literature, but it seems to be interesting for investigators in the future.

## 6. Strengths and Limitations of the Study

The strength of our study is the large size of the sample that consists of consecutive patients from real world clinical practice, which enhances the alignment of the study results with real-life scenarios. In addition, focusing on age-specific groups prevents the influence of age-related differences in the perception of rhinoplasty outcomes. On the other hand, the expectations of older populations following rhinoplasty may differ significantly, highlighting the need for further studies focusing on patients across various age groups.

In turn, as limitations, the disproportionate number of female and male participants should be noted with, 459 (80.5%) women and 111 (19.5%) men. Therefore, future research should aim to include a higher number of male subjects. Additionally, it would be beneficial to evaluate the SF-36 questionnaire outcomes along with the FROI-17 and ROE to allow for more comprehensive assessments. We excluded individuals with a history of depression to minimize its potential influence on the study outcomes. However, given the increasing prevalence of psychological disorders, it seems reasonable to consider conducting a similar study that includes such patients. This transparency not only strengthens the validity of the study’s conclusions but also improves the study’s overall rigor and reproducibility by allowing other researchers to understand the basis for these criteria.

## 7. Conclusions

In summary, using normative data as part of pre-surgical evaluations can provide an objective framework for identifying patients who may not benefit from surgery due to underlying psychological disorders. This approach may help clinicians differentiate between patients who are likely to achieve satisfaction with their surgical outcomes and those for whom surgery may exacerbate mental health issues. Moreover, this information may be valuable for healthcare systems and insurance providers, as it reinforces the importance of psychological screening in achieving successful and sustainable aesthetic outcomes. Ultimately, integrating psychological evaluation with physical assessment ensures a more holistic approach to patient care in aesthetic medicine.

## Figures and Tables

**Table 1:** Outcomes of the Functional Rhinoplasty Outcome Inventory-17 (FROI-17) questionnaires.

	FROI-17			
	All Participants			
	Overall Score	Nasal Symptoms	General Symptoms	Self-Confidence
Mean ± SD	9.1 ± 13.3	4.0 ± 6.0	5.0 ± 7.7	0.6 ± 1.5
Median (Min–Max)	4 (0–72)	1 (0–29)	2 (0–37)	0 (0–10)
	**Women**			
Mean ± SD	9.3 ± 15.3	3.7 ± 6.5	5.3 ± 8.8	0.9 ± 2.0
Median (Min–Max)	4 (0–72)	0 (0–29)	2 (0–37)	0 (0–10)
	**Men**			
Mean ± SD	9.1 ± 12.7	4.0 ± 5.9	4.9 ± 7.4	0.5 ± 1.3
Median (Min–Max)	4 (0–64)	2 (0–27)	2 (0–37)	0 (0–8)

SD, standard deviation.

**Table 2:** Outcomes of the Rhinoplasty Outcome Evaluation (ROE) questionnaires.

	ROE		
	Overall Score	Women	Men
Mean ± SD	13.4 ± 2.3	13.4 ± 2.1	13.4 ± 2.3
Median (Min–Max)	13 (7–24)	14 (7–19)	13 (7–24)

SD, standard deviation.

**Table 3:** Statistical significance of the differences between groups by sex.

	*p*-Value
Overall score ROE	0.932
Overall score FROI-17	0.192
nasal symptoms	0.027 *
general symptoms	0.613
self-confidence	0.425

* Results in bold indicate statistical significance. FROI-17, Functional Rhinoplasty Outcome Inventory-17; ROE, Rhinoplasty Outcome Evaluation.

**Table 4:** Correlation analysis between the Rhinoplasty Outcome Evaluation and Functional Rhinoplasty Outcome Inventory-17 overall scores.

R	*p*-Value
all participants	
−0.413	<0.001
women	
−0.314	0.001
men	
−0.437	<0.001

## Data Availability

The dataset is available on request from the authors.

## Abbreviations

BDD	body dysmorphic disorder
BPD	borderline personality disorder
FROI-17	Functional Rhinoplasty Outcome Inventory 17
QoL	quality of life
ROE	Rhinoplasty Outcome Evaluation
SF-36	Short Form 36 Health Survey
